# Gene-based safety evaluation of molybdenum-containing biomaterials for cardiovascular and cerebrovascular diseases

**DOI:** 10.1016/j.gendis.2025.101516

**Published:** 2025-01-05

**Authors:** Yang Zhang, Yunong Shen, Miaowen Jiang, Guiyou Liu, Hongkang Zhang, Baoying Song, Yufeng Zheng, Xunming Ji, Ming Li

**Affiliations:** a Department of Neurology, Xuanwu Hospital, Capital Medical University, Beijing 100053, China; b China-America Institute of Neurology, Xuanwu Hospital, Capital Medical University, Beijing 100053, China; c School of Materials Science and Engineering, Peking University, Beijing 100871, China; d Beijing Institute for Brain Disorders, Capital Medical University, Beijing 100069, China; e Department of Neurosurgery, Xuanwu Hospital, Capital Medical University, Beijing 100053, China

Molybdenum (Mo)-containing materials are emerging as highly promising biomaterials in intracranial and cardiovascular medicine, attributable to the distinctive properties of metallic Mo, including its superior mechanical strength, electrical conductivity, consistent corrosion behavior, favorable radiopacity, and the functional versatility of Mo-based nanomaterials.^1^ However, their biological safety within the cardiovascular and cerebrovascular domains remains unknown.

Our research aimed to assess the safety implications of heightened serum Mo levels in individuals with cardiovascular and cerebrovascular diseases after the degradation of Mo-containing biomaterials (Fig. 1A). Mendelian randomization (MR) analysis, informed by genome-wide association studies (GWAS), has emerged as a prominent approach to ascertain causality. Utilizing gene data from recent GWAS and the MR analysis method, the study analyzed the causal effect of increasing Mo concentration in serum on cardiovascular and cerebrovascular diseases and found no significant impact of increased serum Mo levels on the occurrence of various cardiovascular and cerebrovascular diseases, including coronary artery disease, heart failure, atrial fibrillation, ischemic stroke, intracranial hemorrhage, and intracranial aneurysm. Our paper provides initial validation regarding the safety of elevated serum Mo levels resulting from biomaterial degradation.

**Figure 1:**
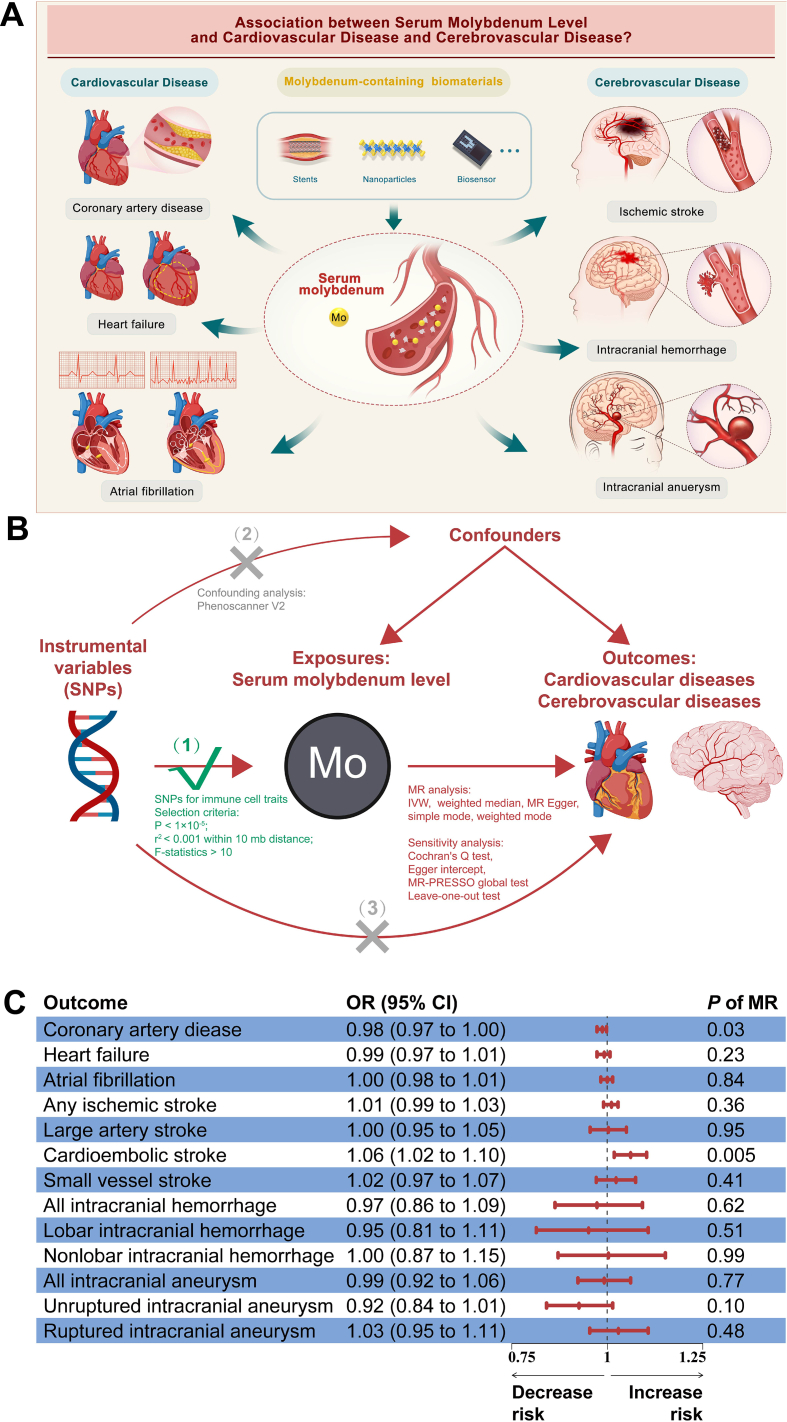
Gene-based safety evaluation of molybdenum-containing biomaterials for cardiovascular and cerebrovascular diseases. **(A)** Conceptual diagram of serum molybdenum levels and cardiovascular and cerebrovascular diseases. **(B)** Mendelian randomization (MR) analysis framework and process flow linking serum molybdenum levels to cardiovascular and cerebrovascular diseases. The model is predicated on these premises: i) instrumental variables (IVs) are associated with serum molybdenum levels; ii) they are unlinked to confounders; iii) any influence of the IVs on cardiovascular and cerebrovascular conditions occurs exclusively via serum molybdenum levels. **(C)** MR analysis results for the association between serum molybdenum levels and cardiovascular and cerebrovascular diseases. CI, confidence interval; IVW, inverse variance weighted; MR, Mendelian randomization; MR-PRESSO, MR Pleiotropy RESidual Sum and Outlier; SNPs, single nucleotide polymorphisms; OR, odd ratio.

This study strictly met three core assumptions for the MR analysis and the overarching design of this study was depicted in Figure 1B. This study also followed the standard MR research report guidelines (STROBE-MR). Some statements and methods could be found in the supplementary information where we also attached the STROBE-MR checklist in.

To meet the three core assumptions, the most important thing is to choose the correct instrumental variables (IVs). First, to identify adequate IVs, we combined the single nucleotide polymorphisms (SNPs) from two datasets on serum Mo levels. In cases of overlap, the SNP with the lower *P*-value was chosen. Due to a scarcity of SNPs meeting the genome-wide significance threshold of 5 × 10^−8^, a locus-wide significance level of *P* < 1 × 10^−5^ was adopted as the criterion for IV selection. SNPs were then clustered to identify independent variants, using a linkage disequilibrium threshold (r^2^) of 0.001 and a minimum distance of 10 Mb between SNPs. F-statistics were calculated for each SNP, with an F-statistic greater than 10 indicating sufficient instrument strength. This step made the IVs satisfy the assumptions of relevance for IVs. Then, SNPs with strong associations with potential confounders were excluded via the Phenoscanner V2 website (http://www.phenoscanner.medschl.cam.ac.uk), which made the IVs satisfy the assumptions of independence for IVs. To achieve the assumptions of exclusion restriction, SNPs were extracted from the outcome datasets, and those closely linked to the outcomes were excluded. Then the SNPs were harmonized, and mismatched as well as palindromic SNPs were discarded. The final set of SNPs was then designated as IVs.

Due to *P* < 0.0038 (after Bonferroni correction) was considered statistically significant, the principal MR analysis indicated that serum Mo levels did not significantly impact cardiovascular and cerebrovascular diseases, as demonstrated by *P*-values post-Bonferroni correction: coronary artery disease (odds ratio/OR = 0.98; 95% confidence interval/CI: 0.97–1.00; *P* = 0.03); heart failure (OR = 0.99; 95% CI: 0.97–1.01; *P* = 0.23); atrial fibrillation (OR = 1.00; 95% CI: 0.98–1.01; *P* = 0.84); ischemic stroke (OR = 1.01; 95% CI: 0.99–1.03; *P* = 0.36); large artery stroke (OR = 1.00; 95% CI: 0.95–1.05; *P* = 0.95); cardioembolic stroke (OR = 1.06; 95% CI: 1.02–1.10; *P* = 0.005); small vessel stroke (OR = 1.02; 95% CI: 0.97–1.07; *P* = 0.41); all intracranial hemorrhage (OR = 0.97; 95% CI: 0.86–1.09; *P* = 0.62); lobar intracranial hemorrhage (OR = 0.95; 95% CI: 0.81–1.11; *P* = 0.51); non-lobar intracranial hemorrhage (OR = 1.00; 95% CI: 0.87–1.15; *P* = 0.99); all intracranial aneurysm (OR = 0.99; 95% CI: 0.92–1.06; *P* = 0.77); unruptured intracranial aneurysm (OR = 0.92; 95% CI: 0.84–1.01; *P* = 0.10); and ruptured intracranial aneurysm (OR = 1.03; 95% CI: 0.95–1.11; *P* = 0.48) (Fig. 1C). Supplementary MR analysis corroborated these findings (Table S1).

For the sensitivity analysis, no heterogeneity or pleiotropy was detected using Cochrane’s *Q* test, the MR-Egger intercept, or the MR-PRESSO global test (Table S2). The stability of the results was further validated through scatter plots, funnel plots, and leave-one-out analysis (Fig. S1–3). In the scatter plots, each point represents a genetic variant, illustrating its relationship with the exposure and outcomes, while the fitted lines for different models clearly depict the association between serum Mo levels and cardiovascular or cerebrovascular diseases. The clustering of genetic variants around the fitted lines, with no noticeable outliers, supports the consistency of the findings. The funnel plots exhibit a funnel-shaped distribution for each group, indicating no heterogeneity. Leave-one-out analysis confirmed that no single SNP significantly influenced the results. Additionally, the statistical power for all outcomes exceeded 80%, highlighting the robustness and reliability of the MR findings. Detailed methods for the MR analysis are provided in the supplementary material. The biosafety of biomaterials is a critical concern. To date, the focus has largely been on the cytotoxicity posed by Mo-containing biomaterials to adjacent tissues during *in situ* biodegradation.^2^ Yet, it is also crucial to consider that corrosion, degradation, and hydrolysis of these biomaterials within the body can elevate serum Mo levels. Given that the cardiovascular and cerebrovascular systems are highly sensitive to serum alterations, changes in Mo levels could potentially lead to pathological changes.

Numerous clinical studies have examined the effects of serum Mo levels on various cardiovascular and cerebrovascular conditions. Guo et al initially reported an inverse relationship between dietary Mo content and the risk of ischemic heart disease and hypertensive heart disease in the Chinese population.^3^ Conversely, Xiao et al found a significant association between increased serum Mo levels and a higher risk of ischemic stroke in the Dongfeng-Tongji cohort study in 2019.^4^ Two years later, Shi et al observed associations between serum Mo levels and both coronary heart disease and stroke, utilizing updated data from the same cohort.^5^

Previous observational studies have shown inconsistent results, likely due to limitations such as confounding factors, challenges with reverse causation, and population-specific variability in disease prevalence. These issues hinder a comprehensive assessment of the relationship between serum Mo levels and cardiovascular or cerebrovascular diseases. To address these shortcomings, we conducted an MR study, which revealed no significant impact of serum Mo levels on the incidence of these diseases.

This analysis is subject to several noteworthy limitations. The initial threshold for selecting IVs was set at a locus-wide significance level of 1 × 10^−5^, which may limit the robustness of the findings. Future studies should aim to increase the sample size of GWAS to identify more reliable IVs for further validation. Moreover, although statistical significance was not achieved, the *P*-value for the potential adverse effects of serum Mo on cardioembolic stroke was below 0.05, highlighting the need for further investigation into this association. Finally, while this study offers preliminary insights at the genetic level, additional validation through *in vitro* and *in vivo* experimental studies is essential to corroborate these findings.

In summary, to the best of our knowledge, this study represents the first investigation into the causal relationship between the degradation of Mo-containing biomaterials and their effects on cardiovascular and cerebrovascular diseases. Preliminary MR analysis indicates that elevated serum Mo levels, resulting from the degradation of these biomaterials, do not exert a significant impact on the incidence or progression of cardiovascular and cerebrovascular diseases. These findings provide further evidence supporting the *in vivo* safety profile of Mo-containing biomaterials.

## Ethics declaration

Ethical approval was waived, and informed consent of participants was obtained previously due to the published GWAS summary statistics. Consent to participate is not applicable in this paper.

## Funding

This study was funded by the 10.13039/501100001809National Natural Science Foundation of China (No. 82027802, 82102220), Research Funding on Translational Medicine from 10.13039/501100009592Beijing Municipal Science and Technology Commission (China) (No. Z221100007422023), 10.13039/501100009331Beijing Hospitals Authority Clinical Medicine Development of Special Funding Support (No. YGLX202325), Non-profit Central Research Institute Fund of Chinese Academy of Medical (No. 2023-JKCS-09), Beijing Association for Science and Technology Youth Talent Support Program (China) (No. BYESS2022081), Beijing Municipal Natural Science Foundation of China (No. 7244510), Science and Technology Innovation Service Capacity Building Project of 10.13039/501100003213Beijing Municipal Education Commission (No. 11000023T000002157177), and Outstanding Young Talents Program of Capital Medical University (China) (No. B2305).

## CRediT authorship contribution statement

**Yang Zhang:** Writing – original draft, Software, Methodology, Investigation, Formal analysis, Conceptualization. **Yunong Shen:** Investigation, Conceptualization. **Miaowen Jiang:** Software. **Guiyou Liu:** Software. **Hongkang Zhang:** Investigation. **Baoying Song:** Investigation. **Yufeng Zheng:** Writing – review & editing, Conceptualization. **Xunming Ji:** Writing – review & editing, Conceptualization. **Ming Li:** Writing – review & editing, Project administration, Methodology.

## Conflict of interests

The authors declared no conflict of interests.

## Data Availability

The datasets analyzed during the current study are available from the corresponding author upon reasonable request.
