# Supplementary material for: Gene-based safety evaluation of molybdenum-containing biomaterials for cardiovascular and cerebrovascular diseases
Source: Genes Dis. 2025 Jan 5;12(5):101516. doi: 10.1016/j.gendis.2025.101516 (PMC12148557; doi:10.1016/j.gendis.2025.101516)
Supplement: Multimedia component 1 [file mmc1.docx]

Supplementary Materials for

**Gene-based safety evaluation** **of** **molybdenum-containing biomaterials for cardiovascular and cerebrovascular diseases**

**This file includes:**

Method

Table S1 to S2

Figure S1 to S3

Method

Data source

The GWAS datasets for serum Mo levels were sourced from two distinct studies: one involving 949 European participants [1] and another with 2,488 Chinese participants [2]. The conditions analyzed in this research are prevalent cardiovascular and cerebrovascular diseases, including CAD, HF, AF, ischemic stroke (IS), intracranial hemorrhage (ICH), and IA. For CAD, a published GWAS meta-analysis provided summary data for 122,733 cases and 424,528 controls [3]. HF-related genetic data were extracted from a recent GWAS meta-analysis, which included 47,309 patients and 930,014 controls [4]. Summary statistics for AF were compiled from five cohort studies, encompassing 60,620 cases and 970,216 controls [5]. The MEGASTROKE collaboration supplied IS data, featuring 40,585 cases and 406,111 controls. For subtyping, patients were categorized into any ischemic stroke (AIS, n = 34,217), large-artery stroke (LAS, n = 4,373), cardioembolic stroke (CES, n = 7,193), and small vessel stroke (SVS, n = 5,386) based on established criteria [6]. ICH summary data were obtained from a GWAS including 1,545 patients (664 lobar and 881 deep) and 1,481 controls [7]. The largest GWAS on IA provided data for 7,495 cases and 71,934 controls, with IA cases further divided into unruptured (N = 2,070) and ruptured (N = 5,140) subtypes [8]. All outcome datasets originated from European cohorts.

Statistical analyses

To assess the relationship between serum Mo levels and cardiovascular and cerebrovascular diseases, the inverse-variance weighted (IVW) method was employed as the primary MR approach due to its robust statistical power [9]. However, IVW presupposes the validity of all instrumental variables, an assumption that may not hold in practical scenarios. Therefore, to ensure the robustness of our causal estimates, additional methods that do not require all genetic variants to be valid IVs were applied. The weighted median approach, effective when a majority of IVs are valid, offers reliable estimates and greater tolerance for invalid IVs [10]. The MR-Egger method, leveraging the Instrument Strength Independent of Direct Effect (InSIDE) criterion, was used to provide unbiased causal effect estimates [11]. Additionally, simple mode and weighted mode analyses were incorporated as supplementary MR methodologies.

Sensitivity analyses were conducted to ascertain the robustness of the findings. These analyses included tests for horizontal pleiotropy and heterogeneity. The IVW method utilized Cochran's *Q* test to assess heterogeneity, indicated by a *P*-value from Cochran's *Q* statistic below 0.05 [12]. To evaluate potential horizontal pleiotropy in the MR framework, the MR-Egger intercept test and the MR-PRESSO (MR-Pleiotropy Residual Sum and Outlier) test were employed. An MR-Egger regression intercept differing from zero suggested the presence of horizontal pleiotropy [11]. Additionally, the global test in MR-PRESSO was used to identify horizontal pleiotropic outliers [13]. Scatter plots, funnel plots, and leave-one-out analyses were generated to enhance interpretability and detect any outliers or heterogeneity.

A statistically significant *P-*value threshold was set at 0.0038 (0.05/13) applying the Bonferroni correction, as the MR analysis involved 13 different outcomes. A relationship was considered suggestive if the P-value fell between the standard significance level (0.05) and the Bonferroni-adjusted threshold. The TwoSampleMR package (version 0.5.6) in the R statistical software (version 4.2.1) was primarily utilized for the analysis.

**Statistical terminology**

1. Locus-wide Significance

In the context of genetic studies, locus-wide significance refers to the statistical threshold used to determine whether a particular genetic variant (often at a specific chromosomal location, or locus) is associated with a trait or disease. It's commonly used in genome-wide association studies (GWAS).

Locus: Refers to a specific location on a chromosome.

Wide: The "wide" part of locus-wide significance means that the test considers multiple loci (locations across the genome) at once, as opposed to focusing on a single gene or a very narrow region.

Significance: Typically involves a p-value threshold, below which a result is considered statistically significant. However, because many tests are done simultaneously across the genome, corrections for multiple comparisons (like the Bonferroni correction) are used to reduce the risk of false positives.

In simpler terms, locus-wide significance is the statistical measure used to determine if a genetic variant at a given locus is likely to be associated with the condition being studied, considering the overall number of tests performed in a study.

2. P-Value

The p-value is a measure used in statistical hypothesis testing to determine the strength of the evidence against the null hypothesis. In simpler terms, it's a way to quantify how likely it is that the observed results were due to chance.

A p-value of 0.05, for example, means that there’s a 5% probability that the results are due to random chance (and thus a 95% chance that the observed effect is real).

If the p-value is less than a certain threshold (often 0.05), the result is considered statistically significant, meaning the observed effect is unlikely to have happened by chance.

3. Confidence Interval (CI)

A confidence interval is a range of values that is used to estimate the true value of a population parameter (like a mean or a regression coefficient), based on sample data. The interval gives a range of plausible values for the parameter.

A 95% confidence interval means that if you were to take 100 different samples and compute the CI for each, 95 of them would contain the true population parameter.

Reference

[1] E. Ng, et al., Genome-wide association study of toxic metals and trace elements reveals novel associations, *Human Molecular Genetics* 24 (16) (2015) 4739-4745, https://doi.org/10.1093/hmg/ddv190.

[2] W. Yang, et al., Genome-wide association and Mendelian randomization study of blood copper levels and 213 deep phenotypes in humans, *Communications Biology* 5 (1) (2022) 405, https://doi.org/10.1038/s42003-022-03351-7.

[3] P. van der Harst, N. Verweij, Identification of 64 Novel Genetic Loci Provides an Expanded View on the Genetic Architecture of Coronary Artery Disease, *Circulation Research* 122 (3) (2018) 433-443, https://doi.org/10.1161/CIRCRESAHA.117.312086.

[4] S. Shah, et al., Genome-wide association and Mendelian randomisation analysis provide insights into the pathogenesis of heart failure, *Nature Communications* 11 (1) (2020) 163, https://doi.org/10.1038/s41467-019-13690-5.

[5] C. Roselli, et al., Multi-ethnic genome-wide association study for atrial fibrillation, *Nature Genetics* 50 (9) (2018) 1225-1233, https://doi.org/10.1038/s41588-018-0133-9.

[6] R. Malik, et al., Multiancestry genome-wide association study of 520,000 subjects identifies 32 loci associated with stroke and stroke subtypes, *Nature Genetics* 50 (4) (2018) 524-537, https://doi.org/10.1038/s41588-018-0058-3.

[7] D. Woo, et al., Meta-analysis of genome-wide association studies identifies 1q22 as a susceptibility locus for intracerebral hemorrhage, *American Journal of Human Genetics* 94 (4) (2014) 511-521, https://doi.org/10.1016/j.ajhg.2014.02.012.

[8] M.K. Bakker, et al., Genome-wide association study of intracranial aneurysms identifies 17 risk loci and genetic overlap with clinical risk factors, *Nature Genetics* 52 (12) (2020) 1303-1313, https://doi.org/10.1038/s41588-020-00725-7.

[9] S. Burgess, et al., Sensitivity Analyses for Robust Causal Inference from Mendelian Randomization Analyses with Multiple Genetic Variants, *Epidemiology* 28 (1) (2017) 30-42, https://doi.org/10.1097/EDE.0000000000000559.

[10] J. Bowden, et al., Consistent Estimation in Mendelian Randomization with Some Invalid Instruments Using a Weighted Median Estimator, *Genetic Epidemiology* 40 (4) (2016) 304-314, https://doi.org/10.1002/gepi.21965.

[11] J. Bowden, et al., Mendelian randomization with invalid instruments: effect estimation and bias detection through Egger regression, *International Journal of Epidemiology* 44 (2) (2015) 512-525, https://doi.org/10.1093/ije/dyv080.

[12] F.D. Greco M, et al., Detecting pleiotropy in Mendelian randomisation studies with summary data and a continuous outcome, *Statistics in Medicine* 34 (21) (2015) 2926-2940, https://doi.org/10.1002/sim.6522.

[13] M. Verbanck, et al., Detection of widespread horizontal pleiotropy in causal relationships inferred from Mendelian randomization between complex traits and diseases, *Nature Genetics* 50 (5) (2018) 693-698, https://doi.org/10.1038/s41588-018-0099-7.

| **Table S1. Supplementary MR analysis results for serum molybdenum level and cardiovascular and cerebrovascular disease.** | | | |
| --- | --- | --- | --- |
| **Outcomes** | **MR method** | **OR (95% CI)** | ***P*** |
| CAD | Weighted median | 0.98 (0.96-1.00) | 0.09 |
|  | MR Egger | 1.00 (0.97-1.02) | 0.79 |
|  | Simple mode | 0.98 (0.94-1.01) | 0.21 |
|  | Weighted mode | 0.99 (0.97-1.02) | 0.49 |
| HF | Weighted median | 1.00 (0.97-1.02) | 0.72 |
|  | MR Egger | 0.99 (0.96-1.03) | 0.75 |
|  | Simple mode | 0.99 (0.96-1.02) | 0.45 |
|  | Weighted mode | 0.99 (0.97-1.02) | 0.51 |
| AF | Weighted median | 0.99 (0.97-1.01) | 0.50 |
|  | MR Egger | 1.01 (0.98-1.05) | 0.59 |
|  | Simple mode | 0.99 (0.95-1.02) | 0.41 |
|  | Weighted mode | 0.99 (0.97-1.02) | 0.49 |
| AIS | Weighted median | 1.00 (0.97-1.03) | 0.93 |
|  | MR Egger | 0.97 (0.93-1.01) | 0.18 |
|  | Simple mode | 1.00 (0.96-1.05) | 0.95 |
|  | Weighted mode | 0.99 (0.96-1.03) | 0.72 |
| LAS | Weighted median | 0.99 (0.93-1.06) | 0.86 |
|  | MR Egger | 0.96 (0.86-1.06) | 0.40 |
|  | Simple mode | 0.98 (0.89-1.09) | 0.75 |
|  | Weighted mode | 0.99 (0.91-1.08) | 0.89 |
| CES | Weighted median | 1.05 (0.99-1.11) | 0.08 |
|  | MR Egger | 1.04 (0.95-1.13) | 0.44 |
|  | Simple mode | 1.04 (0.93-1.16) | 0.47 |
|  | Weighted mode | 1.03 (0.94-1.12) | 0.56 |
| SVS | Weighted median | 0.99 (0.93-1.06) | 0.86 |
|  | MR Egger | 1.01 (0.90-1.13) | 0.89 |
|  | Simple mode | 0.97 (0.88-1.07) | 0.56 |
|  | Weighted mode | 0.99 (0.93-1.07) | 0.86 |
| All ICH | Weighted median | 1.01 (0.85-1.19) | 0.94 |
|  | MR Egger | 0.75 (0.49-1.12) | 0.18 |
|  | Simple mode | 1.05 (0.80-1.37) | 0.75 |
|  | Weighted mode | 1.03 (0.82-1.31) | 0.79 |
| Lobar ICH | Weighted median | 0.95 (0.77-1.18) | 0.64 |
|  | MR Egger | 0.74 (0.43-1.27) | 0.30 |
|  | Simple mode | 1.03 (0.73-1.46) | 0.86 |
|  | Weighted mode | 1.03 (0.77-1.37) | 0.86 |
| Nonlobar ICH | Weighted median | 1.02 (0.84-1.25) | 0.81 |
|  | MR Egger | 0.88 (0.54-1.45) | 0.62 |
|  | Simple mode | 1.04 (0.77-1.40) | 0.80 |
|  | Weighted mode | 1.04 (0.81-1.33) | 0.79 |
| All IA | Weighted median | 1.02 (0.94-1.10) | 0.60 |
|  | MR Egger | 1.18 (0.97-1.44) | 0.12 |
|  | Simple mode | 0.99 (0.86-1.14) | 0.91 |
|  | Weighted mode | 1.07 (0.98-1.17) | 0.17 |
| Unruptured IA | Weighted median | 0.90 (0.79-1.03) | 0.11 |
|  | MR Egger | 1.13 (0.84-1.51) | 0.44 |
|  | Simple mode | 0.87 (0.69-1.10) | 0.26 |
|  | Weighted mode | 0.89 (0.72-1.10) | 0.29 |
| Ruptured IA | Weighted median | 1.07 (0.98-1.17) | 0.15 |
|  | MR Egger | 1.22 (0.98-1.51) | 0.10 |
|  | Simple mode | 1.07 (0.92-1.24) | 0.38 |
|  | Weighted mode | 1.12 (1.00-1.25) | 0.07 |
| CAD, coronary artery disease; HF, heart failure; AF, atrial fibrillation; AIS, any ischemic stroke; LAS, large artery stroke; CES, cardioembolic stroke; SVS, small vessel stroke; ICH, intracerebral hemorrhage; IA, intracranial aneurysm; MR, Mendelian randomization; OR, odd ratio; CI, confidence interval. | | | |

| **Table S2 Sensitivity analysis results for serum molybdenum level and cardiovascular and cerebrovascular disease.** | | | | | |
| --- | --- | --- | --- | --- | --- |
| **Outcomes** | **Heterogeneity test** | | **Pleiotropy test** | | |
|  | **Q−statistic** | ***P*** | **MR-Egger** | | ***P* for MR−PRESSO global test** |
|  |  |  | ***intercept*** | ***P*** |  |
| CAD | 23.30 | 0.39 | 0.004 | 0.34 | 0.38 |
| HF | 28.50 | 0.16 | 0.01 | 0.82 | 0.19 |
| AF | 23.94 | 0.30 | 0.01 | 0.48 | 0.34 |
| AIS | 15.89 | 0.82 | 0.01 | 0.06 | 0.82 |
| LAS | 16.59 | 0.79 | 0.02 | 0.34 | 0.80 |
| CES | 25.55 | 0.27 | 0.01 | 0.61 | 0.26 |
| SVS | 28.28 | 0.17 | 0.02 | 0.80 | 0.19 |
| All ICH | 13.49 | 0.45 | 0.06 | 0.21 | 0.50 |
| Lobar ICH | 11.62 | 0.64 | 0.08 | 0.37 | 0.64 |
| Nonlobar ICH | 13.93 | 0.46 | 0.08 | 0.61 | 0.47 |
| All IA | 22.19 | 0.05 | 0.03 | 0.08 | 0.05 |
| Unruptured IA | 10.01 | 0.69 | 0.04 | 0.18 | 0.62 |
| Ruptured IA | 17.89 | 0.16 | 0.03 | 0.13 | 0.16 |
| CAD, coronary artery disease; HF, heart failure; AF, atrial fibrillation; AIS, any ischemic stroke; LAS, large artery stroke; CES, cardioembolic stroke; SVS, small vessel stroke; ICH, intracerebral hemorrhage; IA, intracranial aneurysm; MR, Mendelian randomization; MR-PRESSO, MR Pleiotropy RESidual Sum and Outlier. | | | | | |



**Fig. S1. Scatter plots from genetically predicted serum molybdenum level on cardiovascular and cerebrovascular diseases.** The lines indicate the estimate of effect using IVW, MR-Egger, Simple mode, Weighted median, and Weighted mode. The circles represent the marginal genetic associations between serum molybdenum levels and the risk of each outcome for individual variants, with error bars depicting 95% CIs. SNP, single nucleotide polymorphism; CAD, coronary artery disease; HF, heart failure; AF, atrial fibrillation; AIS, any ischemic stroke; LAS, large artery stroke; CES, cardioembolic stroke; SVS, small vessel stroke; ICH, intracerebral hemorrhage; IA, intracranial aneurysm.



**Fig. S2.** **Funnel plots from genetically predicted serum molybdenum levels on cardiovascular and cerebrovascular diseases.** MR, Mendelian randomization; CAD, coronary artery disease; HF, heart failure; AF, atrial fibrillation; AIS, any ischemic stroke; LAS, large artery stroke; CES, cardioembolic stroke; SVS, small vessel stroke; ICH, intracerebral hemorrhage; IA, intracranial aneurysm.



 **Fig. S3. Leave-one-out analysis from genetically predicted serum molybdenum level on cardiovascular and cerebrovascular diseases.** CAD, coronary artery disease; HF, heart failure; AF, atrial fibrillation; AIS, any ischemic stroke; LAS, large artery stroke; CES, cardioembolic stroke; SVS, small vessel stroke; ICH, intracerebral hemorrhage; IA, intracranial aneurysm.
